# Mapping of quantitative trait loci for tuber starch and leaf sucrose contents in diploid potato

**DOI:** 10.1007/s00122-015-2615-9

**Published:** 2015-10-14

**Authors:** Jadwiga Śliwka, Dorota Sołtys-Kalina, Katarzyna Szajko, Iwona Wasilewicz-Flis, Danuta Strzelczyk-Żyta, Ewa Zimnoch-Guzowska, Henryka Jakuczun, Waldemar Marczewski

**Affiliations:** Plant Breeding and Acclimatization Institute, National Research Institute, Młochów, Platanowa 19, 05-831 Młochów, Poland

## Abstract

*****Key message***:**

**Most QTL for leaf sucrose content map to positions that are similar to positions of QTL for tuber starch content in diploid potato.**

**Abstract:**

In the present study, using a diploid potato mapping population and Diversity Array Technology (DArT) markers, we identified twelve quantitative trait loci (QTL) for tuber starch content on seven potato chromosomes: I, II, III, VIII, X, XI, and XII. The most important QTL spanned a wide region of chromosome I (42.0–104.6 cM) with peaks at 63 and 84 cM which explained 17.6 and 19.2 % of the phenotypic variation, respectively. ADP-glucose pyrophosphorylase (AGPase) is the key enzyme for starch biosynthesis. The gene encoding the large subunit of this enzyme, *AGPaseS*-*a*, was localized to chromosome I at 102.3 cM and accounted for 15.2 % of the variance in tuber starch content. A more than 100-fold higher expression of this gene was observed in RT-qPCR assay in plants with the marker allele AGPaseS-a_1334_. This study is the first to report QTL for sucrose content in potato leaves. QTL for sucrose content in leaves were located on eight potato chromosomes: I, II, III, V, VIII, IX, X and XII. In 5-week-old plants, only one QTL for leaf sucrose content was detected after 8 h of darkness; four QTL were detected after 8 h of illumination. In 11-week-old plants, 6 and 3 QTL were identified after dark and light phases, respectively. Of fourteen QTL for leaf sucrose content, eleven mapped to positions that were similar to QTL for tuber starch content. These results provide genetic information for further research examining the relationships between metabolic carbon molecule sources and sinks in potato plants.

**Electronic supplementary material:**

The online version of this article (doi:10.1007/s00122-015-2615-9) contains supplementary material, which is available to authorized users.

## Introduction

In plants, photosynthetic tissues are the main source of net carbon fixed in the Calvin-Benson cycle. Triose-phosphate, the product of carbon assimilation, is converted to transitory starch in the chloroplast or transported to the cytosol, where sucrose biosynthesis takes place. Transitory starch is remobilized into sugars during the night. Sucrose, being the major product of starch degradation, is exported from leaves to sink organs for storage, maintenance, and growth (Geigenberger 2011; Stitt and Zeeman 2012; Bahaji et al. 2014).

In growing potato (*Solanum tuberosum* L.) tubers, starch biosynthesis is the dominant pathway of carbohydrate metabolism (Geigenberger et al. 2004). It is under control of many genetic and environmental factors. Quantitative trait loci (QTL) mapping provides valuable information in terms of the minimal number and approximate genomic position of the factors controlling a complex trait (Chen et al. 2001). QTL analysis requires phenotypic evaluation, molecular profiling, and statistical analysis of a segregating population. In potato, the first QTL analyses of specific gravity, an estimation of starch content, were conducted using diploid populations and isozyme markers (Freyre and Douches 1994a). The resultant linkage map was supplemented with restriction fragment length polymorphism (RFLP) and random amplified polymorphic DNA (RAPD) loci (Freyre and Douches 1994b). Other RFLP linkage maps (Schäfer-Pregl et al. 1998) were supplemented with cleaved amplified polymorphic sequence (CAPS) and sequence characterized amplified region (SCAR) markers to produce a more precise QTL analysis of tuber starch content (TSC) (Chen et al. 2001). Recently, a diploid potato map consisting of amplified fragment length polymorphism (AFLP), simple sequence repeat, and CAPS/SCAR markers was used for QTL analysis of a number of starch characteristics, including starch content (Werij et al. 2012). The quantitative characteristics of this trait were confirmed in the cultivated potato. Tubers from a tetraploid potato population were submitted to linkage mapping and QTL analysis of specific gravity (McCord et al. 2011). QTL for starch content were also evaluated in a series of association mapping experiments conducted in populations of tetraploid breeding clones and cultivars (Li et al. 2008, 2010, 2013).

Potato tubers are strong sink organs, making the potato a suitable model plant in which to study source-sink interactions (Jonik et al. 2012). Pull-approaches aimed at increasing sink-strength and push-approaches intended to enhance source capacity have been distinguished (Flügge et al. 2011), but it remains unclear whether net carbon accumulation is sink- or source-limited in potato (Ferreira et al. 2010). For example, measurements of carbon flux suggest that starch biosynthesis in tubers depends largely on reactions that take place within leaves, with sucrose then being transported from leaf cells to the phloem (Sweetlove et al. 1998; Sweetlove and Hill 2000). A transgenic manipulation that elevated sink strength was shown to increase tuber starch content and yield (Zhang et al. 2008). Furthermore, Jonik et al. (2012) found recently that transgenic enhancement of both source and sink capacity could double tuber starch yield. Conversely, increasing sucrose transport to tubers did not affect starch content in tubers (Leggewie et al. 2003).

Although transitory starch catabolism in leaves is a fairly well characterized metabolic network, the light/dark regulation of this pathway is not clear (Hofius and Börnke 2007). Pull and push approaches can be combined to increase sink and sources capacities and thereby improve crop yields (Flügge et al. 2011). Sucrose is the only carbon metabolite transported from leaves to tubers (Sweetlove et al. 1998) and it appears to be in constant transport (Sweetlove and Hill 2000). However, higher foliar levels of sucrose (Ferreira et al. 2010) and greater sucrose export from leaves during daylight hours relative to dark hours (Geigenberger and Stitt 2000; Ferreira et al. 2010) have been observed. Šimko et al. (1999) found three QTL related to sucrose content in phloem exudate collected from leaves at the end of the light period.

In the current study, we conducted QTL analyses of tuber starch content and leaf sucrose content in diploid potato using Diversity Array Technology (DArT) markers in our linkage map construction. Because tuber development involves complex metabolic changes in the potato plant (for review see, Hannapel 2007), we conducted QTL mapping for sucrose content in leaves before and during the tuber initiation stage. We report the first QTL map for leaf sucrose content in potato after 8 h of darkness and after 8 h of light.

## Materials and methods

### Plant materials

Diploid potato clones (seed parent DG 00-683, pollinator DG 08-28/13) and their F1 progeny (population 12–3, *N* = 183) were used for DArT map construction and QTL analyses. Both parents were interspecific *Solanum* hybrids having *S. tuberosum*, *S. chacoense*, *S. gourlayi*, *S. yungasense*, *S. verrucosum* and *S. microdontum* in their pedigree. The theoretical contributions of *S. tuberosum* in the origin of DG 00-683 and DG 08-28/13 were 64 and 69 %, respectively. The corresponding values for *S. chacoense* were 28 and 11 %. In addition, DG 08-28/13 contains 9 % contribution of *S. phureja* genome. The parental clones were generated within the diploid potato breeding program at the Plant Breeding and Acclimatization Institute—National Research Institute, Młochów, Poland.

### Phenotypic analysis

Tuber starch content (TSC) of the parental clones and of their 12–3 progeny was evaluated in three subsequent vegetation seasons: 2012 (seedlings), 2013 (first tuber generation), and 2014 (second tuber generation). In each of these 3 years, tubers were sprouted for 7–10 days in the sprouting chamber and then planted into foil tunnels for 18 weeks (from May to October). The plants were watered regularly, fertilized, and protected against insects and pathogens. Tuber starch content was estimated from the ratio of tuber weight in air (g) to tuber weight in water (g) as described by Lunden (1956). In 2013 and 2014, F1 individuals were grown in three replications in a random pattern and scored for tuber starch content directly after being harvested.

In 2013, two independent tests were performed for leaf sucrose content (LSC). The parental and F1 plants were grown in pots in a greenhouse. Three-week-old plants were transferred to growth chambers (16-h day/8-h night, 23 °C day/15 °C night, light intensity above the canopy = 4000 Lux). In each test, experiments were carried out with 5-week-old plants before stolon growth and with 11-week-old plants during tuber development. Three terminal leaflets were collected from the uppermost leaves of each plant after 8 h of darkness (dark phase) and after 8 h of illumination (light phase). Samples of three leaflets (0.1 g) were ground in liquid nitrogen and their contents were extracted with 80 % ethanol at 80 °C. Sucrose concentration was measured using a sucrose determination kit (K-SURFG, Megazyme) equipped with β-fructosidase and hexokinase according to the manufacturer’s protocol. Abbreviations of the tested traits are defined in Table 1.

**Table 1:** Phenotypic distributions of tuber starch and leaf sucrose contents in population 12–3

Trait^a^	DG 00-683	DG 08-28/13	*N*	Population mean	Range of variation	*W* value^b^
TSC	20.8 (3.5)	11.8 (0.1)	183	16.0 (2.0)	11.6–22.2	0.986
LSC5AN	3.1 (0.3)	1.2 (0.1)	137	1.2 (0.6)	0.4–3.4	0.852*
LSC5AL	9.0 (2.8)	3.3 (1.2)	137	2.6 (1.2)	0.8–6.4	0.934*
LSC11AN	4.9 (0.1)	4.6 (0.5)	135	3.6 (1.7)	1.0–10.4	0.922*
LSC11AL	11.8 (4.5)	7.4 (1.9)	133	6.1 (3.0)	1.7–15.5	0.908*

Standard deviations are in parentheses

*N* no. F1 individuals analyzed per trait, *TSC* tuber starch content evaluated in years 2012–2014 (% FW), *LSC5AN* leaf sucrose content after 8 h of darkness (mg g^−1^ FW) in 5-week-old plants before stolon growth, *LSC5AL* mean leaf sucrose content after 8 h of light (mg g^−1^ FW) in 5-week-old plants before stolon growth, *LSC11AN* leaf sucrose content after 8 h of darkness (mg g^−1^ FW) in 11-week-old plants during tuber development, *LSC11AL* mean leaf sucrose content after 8 h of light (mg g^−1^ of FW) in 11-week-old plants during tuber development, *LSC* values are means of two experiments performed in 2013

* Deviated from normality at *P* < 0.05

^a^Pearson’s correlation coefficients between tests were significant

^b^Shapiro-Wilk test

### Genetic mapping and QTL analysis

Genomic DNA was isolated from freeze-dried leaf tissue using a GenElute Plant Genomic DNA Miniprep kit (Sigma-Aldrich, St. Louis, MI). The DArT analysis was performed in Diversity Array Pty Ltd. Canberra, Australia, as described for *S. michoacanum* and *S. ruiz*-*ceballosii* by Śliwka et al. (2012a, b), following protocols developed for other plant species (Jaccoud et al. 2001; Wenzl et al. 2004; Akbari et al. 2006). The CAPS markers used are listed in Supplementary Table S1. Linkage analysis was performed with using JoinMap^®^ 4 as described elsewhere (Van Ooijen 2006) with the following settings: CP population type (creating maternal and paternal linkage maps first and then creating a common map), independence LOD as the grouping parameter (significance cut-off, LOD score >3), regression mapping algorithm, and Haldane’s mapping function (Śliwka et al. 2012a). The linkage groups obtained were oriented and named (chromosomes I–XII) by comparison with DArT maps of related species (Śliwka et al. 2012a, b; Sharma et al. 2013). QTL interval mapping was performed in MapQTL^®^ 6 software (Van Ooijen 2009). QTL with an LOD score >3 were considered to be significant.

### *AGPaseS*-*a* expression assay

Expression of *AGPaseS*-*a* was studied for the parents (DG 00-683 and DG 08-28/13) and 20 F1 individuals using a reverse transcription quantitative polymerase chain reaction (RT-qPCR). Total RNA was extracted from tuber samples collected after harvest in 2012 and frozen in liquid nitrogen as described by Chomczyński and Sacchi (1987). The quality of total RNA was evaluated spectrophotometrically and by gel electrophoresis. First-strand cDNA was synthesized from 500 ng of total RNA using random hexamers and RevertAid reverse transcriptase (RT) purchased from Thermo Scientific (Fermentas). Two high- and two low-starch content bulks were prepared. For each bulk, equal amounts of cDNA from the tubers of five plants were mixed. Bulk I included samples from plants with a starch content in the range of 19.0–19.8 %, all of which had the AGPaseS-a_1334_ marker allele. This marker accounted for 15.2 % of the variance in tuber starch content. Bulks II and III source plants had starch contents in the 19.8–21.7 % and 9.9–10.5 % ranges, respectively; only one in five samples in bulks II and III had the AGPaseS-a_1334_ marker. Finally, bulk IV was made up of samples from plants with a starch content in the range of 9.9–12 %, and none of the constituent plants had the AGPaseS-a_1334_ marker. Quantitative RT-qPCR reactions were performed in 96-well plates with a LightCycler 480 II system (Roche, Switzerland). The primer sequences used to detect *AGPaseS*-*a* (GenBank accession X61187) expression were: forward 5′-TCATGATGGGAGCAGACTCCTACC-3′ and reverse 5′-CTTTCCTATCTTTGCGTTCTTGT-3′. Each PCR was carried out in a 20-μl volume containing 1× SYBR Green PCR Master Mix (Roche, Switzerland), 1 μl of cDNA corresponding to 50 ng of total RNA, and 200 nM of each forward and reverse primer. Potato *α*-*tubulin* was used as the reference gene (Śliwka et al. 2013). Thermal cycling conditions were: 4 min denaturation at 95 °C followed by 55 cycles of 10 s at 90 °C, 20 s at 62 °C, and 30 s at 72 °C. PCR product melting point was determined in the range of 65–97 °C and the melting curve was analysed to confirm amplification of gene-specific products. Four technical replicates of each reaction were performed. Relative expression levels were calculated in Microsoft Excel 2010. Statistical analyses, namely *t* tests for ΔΔ*C*_t_ cycle threshold values (Schmittgen and Livak 2008) and calculation of standard errors of the means (SEs), were performed with Statistica software (Stat Soft Inc.).

### *AGPaseS*-*a* cloning and sequencing

The restriction patterns of the AGPaseS-a amplicons obtained after *Taq*I digestion in the F1 individuals indicated that the parents DG 00-683 and DG 08-28/13 were heterozygous and homozygous for this locus (data not shown). The 1334-bp-long fragment uncut by the restriction enzyme in DG 00-683 and the corresponding *Taq*I-untreated PCR product amplified in DG 08-28/13 were extracted from an agarose gel using GenElute™ Gel Extraction Kit (Sigma–Aldrich, St. Louis, MO, USA) and cloned using CloneJET PCR Cloning Kit (Thermo Fisher Scientific Waltham, MA USA). Four plasmids for each cloned sequence were sent to Genomed^®^, Warsaw, Poland, for sequencing. Sequences were analysed using the Lasergene 6.1 software (DNASTAR Inc., Madison, WI, USA).

## Results

### Evaluation of tuber starch content and sucrose content in leaves

Tuber starch content (TSC) and leaf sucrose content (LSC) values of the parents and progeny of population 12–3 are presented in Table 1. TSC values were normally distributed in the population 12–3 plants, except for in the year 2014, whereas LSC values deviated significantly from normality.

### DArT map

Of the 3343 DArT markers scored in population 12–3, 2241 DArT markers segregated (i.e. present in >10 % and <90 % of progeny individuals). Markers with more than 10 % of their data points missing and those with unknown origins (parental clones data missing) were excluded from further analysis. Further markers were removed on the basis of DArT quality parameters as follows: *P* < 60 (37 markers), and call rate <85 (31 markers). Markers with identical patterns of segregation were omitted by the JoinMap^®^ 4 program. The final genetic map contained 1597 markers, including 1584 DArT, 11 CAPS, one SCAR, and one phenotypic (purple flower color) marker. Of these 1596 markers, 492 originated from parent DG 00-683, 405 originated from DG 08-28/13, and 700 descended from both parents. Total length of the map reached 1117 cM. The quantity of markers located on particular chromosomes varied from 86 on chromosome V to 230 on chromosome I. Chromosome length ranged from 66 to 143 cM (Supplementary Fig. S1).

### QTL analysis

**Fig. 1:**
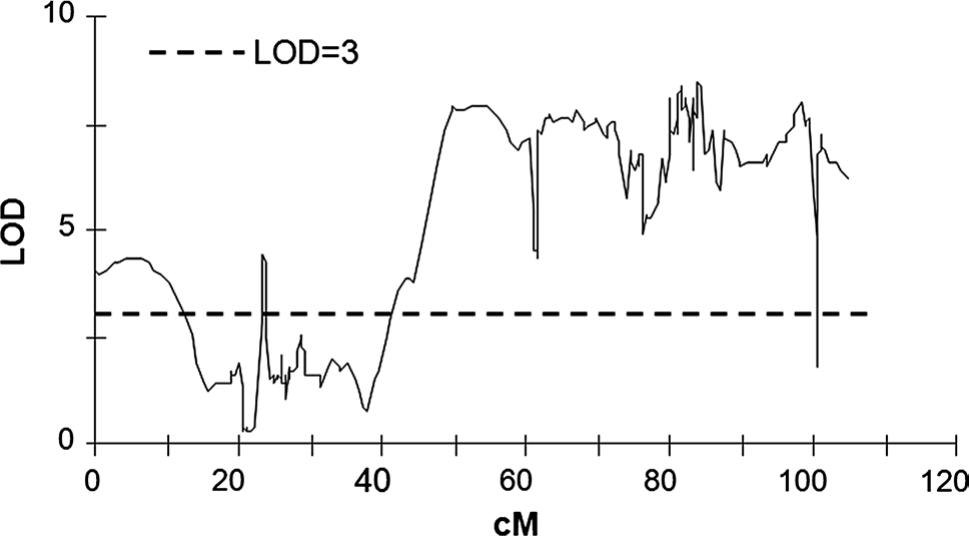
QTL on chromosome I for mean (2012–2014) tuber starch content (TSC) detected in population 12–3. QTL were determined by interval mapping and MapQTL^®^ 6 software (Van Ooijen 2009). Genetic distance in cM is shown on the *x*-axis and QTL effect strength, as indexed by LOD score, is shown on the *y*-axis. The threshold LOD score (LOD = 3) is indicated by a *horizontal line*

QTL for tuber starch content in population 12–3 plants were detected on seven potato chromosomes: I, II, III, VIII, X, XI and XII. Most were significant in all four datasets (TSC12–TSC14 in Supplementary Table S2 and mean TSC in Table 2), indicating that this trait was stable across different vegetation seasons. The QTL on chromosomes III and XII were the exceptions being detected only in the TSC12, TSC14, and mean TSC datasets. The effect on TSC varied from 19.2 % of the variance explained by the QTL on chromosome I (LOD, 8.48) to 9.4 % by the QTL on chromosome III (LOD, 3.91). Marker alleles affecting the trait descended from DG 00-683 (QTL on chromosomes X and XII), DG 08-28/13 (QTL on chromosomes II and III), or both parents (remaining QTL). The most important QTL for mean tuber starch content was located on chromosome I (as presented in detail in Fig. 1), spanning a wide region of the chromosome, from 42.0 to 104.6 cM, with its peak at 84 cM (Fig. 1; Table 2). The variance in tuber starch content attributable to this QTL ranged from 13.0 % (TSC12) to 18.8 % (TSC13), depending on the year (Supplementary Table S2). An effect of similar strength was detected at 63.0 cM. Another minor QTL for tuber starch content was detected at the top end of chromosome I (0–12.3 cM). It explained up to 10.2 % (LOD, 4.29) of the variance in tuber starch content.

**Table 2:** QTL detected for tuber starch content (TSC, means 2012–2014) and leaf sucrose content (LSC) measured after 8 h of darkness (AN) or light (AL) in 5- and 11-week-old plants of the diploid potato population 12–3

Chromosome	Marker at peak or markers flanking virtual peak interval	Marker origin^a^	Trait	Peak position (cM)	LOD	*R* ^2^ (%)	Significant interval (cM)
I	pPt-535988 –pPt-538127	P2, H	LSC5AN	3.5	4.59	14.3	0–13.2
			LSC5AL		7.49	22.3	0–15.6
			TSC		4.29	10.2	0–12.3
	pPt-650366	P2	LSC5AL	26.1	5.60	17.2	22.2–49.0 61.3–67.6
	pPt-535462	P1	LSC11AN	57.5	3.97	12.7	57.5–71.5
	capPt-673196	P1	LSC11AL	63.0	4.85	15.5	57.5–72.8
	toPt-440651	H	TSC	84.0	8.48	19.2	42.0–104.6
II	pPt-656098	P2	LSC11AN	56.0	4.01	12.8	55.9–56.0
	pPt-539763	P2	TSC	55.1	4.35	10.4	35.0–56.0
III	toPt-437014–pPt-538033	P2	TSC	68.1	3.91	9.4	65.1–72.1
V	pPt-656044	P2	LSC11AN	23.0	5.05	15.8	22.0–25.5
VIII	pPt-652452	H	LSC11AL	32.8	3.08	10.1	31.6–32.8
	pPt-656209	P1	LSC11AN	34.8	3.31	10.7	34.8
	toPt-438845	H	TSC	39.6	6.46	14.9	22.8–39.6
IX	pPt-657409	P1	LSC5AL	51.9	3.71	11.7	51.7–51.9
	pPt-537372	P2	LSC11AL	52.9	3.40	11.1	52.9
X	pPt-651091	P1	LSC5AL	24.5	3.36	10.7	24.3–25.5
	pPt-552383	H	LSC11AN	27.1	3.07	9.9	27.1
	pPt-533878	P1	TSC	28.8	6.58	15.3	16.5–46.5
XI	pPt-471789	H	TSC	54.1	5.04	11.9	49.8–55.8
XII		P2	LSC11AN	46.3	4.45	14.1	38.5–49.6
	pPt-656237	P1	TSC	143.5	5.49	12.9	117.6–143.5

Interval mapping of QTL was performed using MapQTL ^®^ 6 (Van Ooijen 2009)

^a^P1- inherited from DG 00-683; P2- inherited from DG 08-28/13; H- descending from both parents

**Fig. 2:**
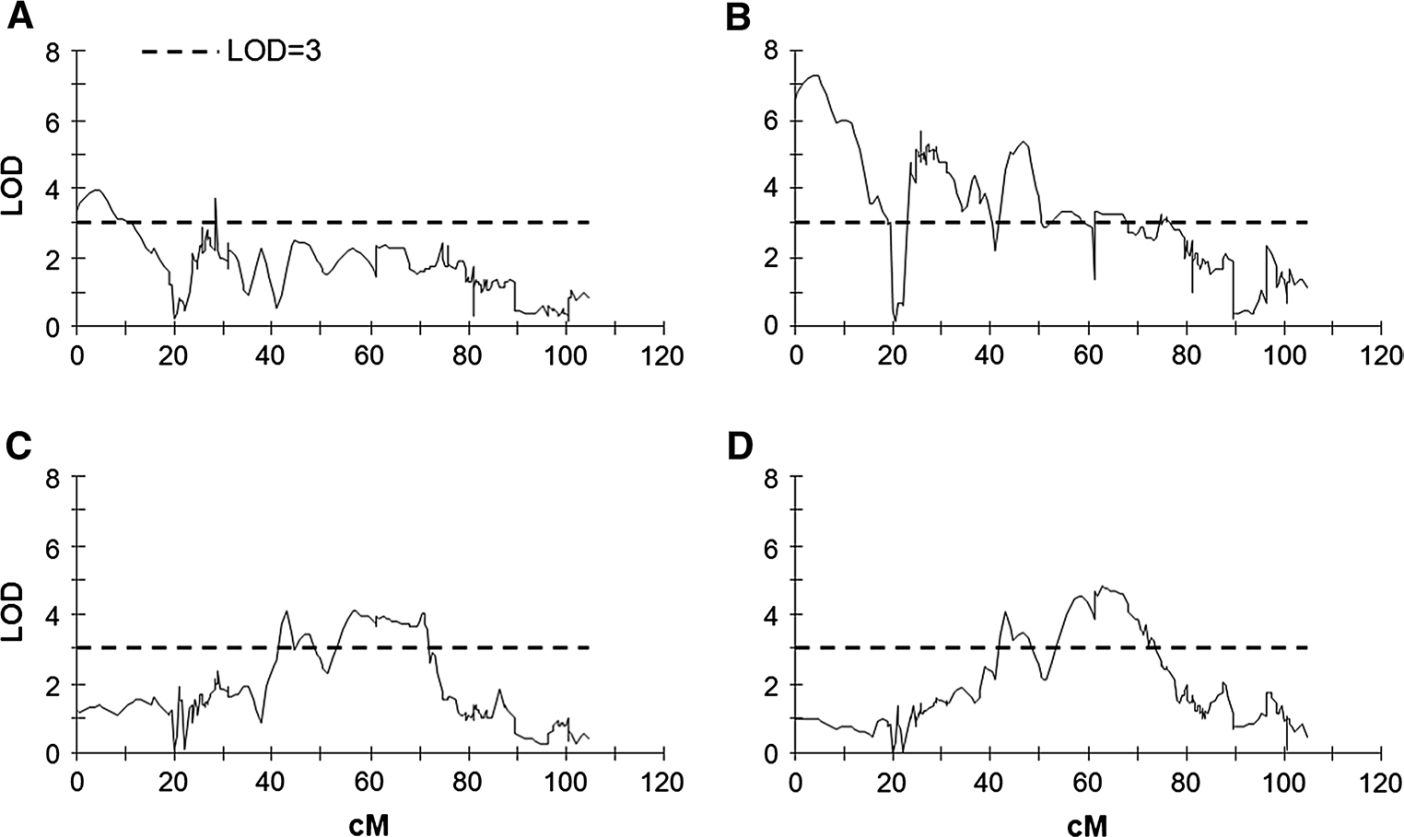
QTL on chromosome I for leaf sucrose content detected in population 12-3. QTL were determined by interval mapping and MapQTL^®^ 6 software (Van Ooijen 2009). Sucrose content was measured in four experimental settings: LSC5AN, after 8 h of night in 5-week-old plants (**a**); LSC5AL, after 8 h of light in 5-week-old plants (**b**); LSC11AN, after 8 h of night in 11-week-old plants (**c**); LSC11AL, after 8 h of light in 11-week-old plants (**d**). Genetic distance (in cM) is shown on the *x*-axis and QTL effect strength, as indexed by LOD score, is shown on the *y*-axis. The threshold LOD score (LOD = 3) is indicated by a *horizontal line*

QTL for leaf sucrose content were detected on eight potato chromosomes: I, II, III, V, VIII, IX, X, and XII. Again, both parents contributed alleles that were significant for these traits (Table 2). Particular QTL were detected in as few as one to as many of three out of four datasets: LSC5AN, LSC5AL, and LSC11AN in different combinations. The most prominent QTL for leaf sucrose content in 5-week-old plants was detected on chromosome I (0–15.6 cM) (Fig. 2a, b) and had a significant effect both after night and after light: LSC5AN (LOD 4.59; *R*^2^ 14.3 %) and LSC5AL (LOD 7.49; *R*^2^ 22.3 %). It overlapped with a minor QTL for tuber starch content detected in all TSC datasets at the top end of chromosome I (0–12.3 cM, *R*^2^ 10.2 %, LOD 4.29) (Fig. 1). Weaker QTL for leaf sucrose content in 5-week-old plants after light were detected on chromosomes I (22.2–49.0 cM), IX, and X (Table 2). A QTL on chromosome I (63.0 cM) also affected leaf sucrose content in tuberizing plants (LSC11AN, LOD 3.97; *R*^2^ 12.7 %) (Table 2, Fig. 2c); this locus was the peak of the most important QTL for LSC11AL (LOD 4.85; *R*^2^ 15.5 %) (Table 2, Fig. 2d) and had a highly significant influence on tuber starch content in all TSC datasets (Supplementary Table S2). The localizations of the significant effects overlapped as follows: LSC5AL, 61.3–67.6 cM; LSC11AN, 57.5–71.5 cM; LSC11AL, 57.5–72.8 cM; and TSC 42.0–104.6 cM (Figs. 1, 2).

An important QTL for leaf sucrose content in tuberizing plants after the dark period was identified on chromosome V (LOD 5.05; *R*^2^ 15.8 %); it affected this trait exclusively (Table 2). Other QTL for leaf sucrose content in 11-week-old plants overlapped to varying extents with QTL for other traits and were located on chromosomes II (LSC11AN, but also TSC), VIII (LSC11AN, LSC11AL, and TSC), IX (LSC11AL and LSC5AL), X (LSC11AN, LSC5AL, and TSC), and XII (LSC11AN) (Table 2).

**Fig. 3:**
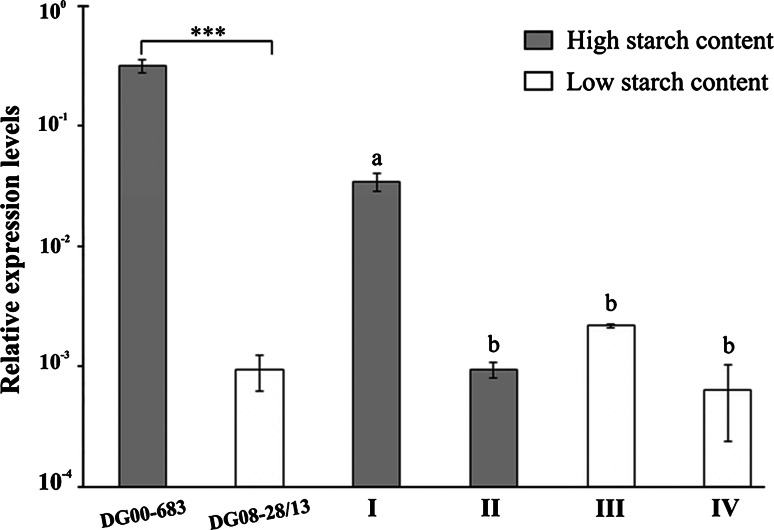
Relative expression of the gene *AGPaseS*-*a* in potato tubers from DG 00-683 and DG 08-28/13 parent plants and progeny bulks I–IV. Mean tuber starch contents for DG 00-683 and DG 08-28/13 were 20.8 and 11.8 %, respectively. Expression is reported in relation to that of the reference gene α-tubulin and shown in log scale. In the samples used for preparation of the bulks, these values were as follows: bulk I, 19.0–19.8 %; bulk II, from 19.8 to 21.7 %; bulk III, 9.9–10.5 %; bulk IV, 9.9–12.0 %. Presence/absence of the marker AGPaseS-a_1334_ in F1 individuals of the bulked-DNA samples: 5/0 (bulk I), 1/4 (bulks II and III), 0/5 (bulk IV). Data represent means of four replications ± SE. *P* < 0.001 (*t* test)

Twelve sequence-specific markers derived from genes involved in sugar metabolism were mapped in population 12–3. Their genetic positions and significance for the tested traits (LOD, *R*^2^), as well as physical positions in the potato DM1-3 genome (v4.03) are given in Supplementary Table S1. Two of these markers, NL-AAM and GPT, mapped to other locations than the locations of the sequences on the basis of which they were designed. Five sequence-specific markers were located within QTL for tuber starch content and/or leaf sucrose content (Supplementary Table S1). For leaf sucrose content, only one sequence-specific marker had a significant effect, NL-AAM. NL-AAM was located within a QTL on chromosome I and explained 15.9 % of the leaf sucrose content variation in the LSC5AL dataset. Four genetic markers β–Amyl, AOX1a-1, AOX1a-2, and AGPaseS-a explained 12.8, 11.0, 12.9, and 15.2 % of the tuber starch content variation observed, respectively (Supplementary Table S1). AGPase (ADP-glucose pyrophosphorylase) is the key enzyme for starch biosynthesis. In our study, the locus *AGPaseS*-*a* was positioned in the most important QTL for tuber starch content on chromosome I at 102.3 cM (Fig. 1). The AGPaseS-a_1334_ amplicon generated form the parent DG 00-683 (GenBank accession no. KT341038) shared 89 % sequence identity with the reference sequence *Lycopersicon esculentum* ADPglucose pyrophosphorylase large subunit gene (DQ322683.1) and 96 % of sequence identity with the genomic sequence of AGPase in the DM1-3 genome (chr01:86093259.0.86094571, v4.03). There was a 8 % sequence difference in single-nucleotide substitutions and short insertion/deletion polymorphisms between AGPaseS-a_1334_ generated form the parent DG 00-683 and the corresponding marker AGPaseS-a_1379_ (KT341039) produced form the parental clone DG 08-28/13. We applied the RT-qPCR technique to show that *AGPaseS*-*a* (amplicon size: 137 bp) exhibited significantly higher expression in the high tuber starch content parent DG 00-683 with the CAPS marker AGPaseS-a_1334_ than in the low tuber starch content parent DG 08-28/13 without AGPaseS-a_1334_ (Fig. 3). Expression of this gene was more than 100-fold higher in progeny bulk I (marker allele AGPaseS-a_1334_ amplified for all five DNA samples) than in progeny bulks II and III (marker allele AGPaseS-a_1334_ present in 1 of 4 bulked-DNA samples), or bulk IV (AGPaseS-a_1334_ absent). The significance of these differences was confirmed by Tukey’s test (Fig. 3).

## Discussion

During the day, sucrose synthesized in photosynthetic tissues is exported to the other parts of the plant to support metabolism, storage, and growth. At night, the plant becomes a net consumer of fixed carbon (Bläsing et al. 2005). In Arabidopsis, sucrose levels in leaves at the end of light period are higher than after the night period (Sun et al. 2011). A similar observation was reported in potato cultivar Solara (Ferreira et al. 2010). In the diploid clones DG 00-683 and DG 08-28/13, leaf sucrose content is also increased in the light period. Higher sucrose levels in the parents of the mapping population during tuber development than in the young plants may reflect a higher requirement for carbon skeletons for all organic compounds, including storage carbohydrates, in the heterotrophic cells.

Five and nine QTL for sucrose content were detected in our 5- and 11-week-old plants, respectively. In 5-week-old plants after darkness, only one QTL, LSC5AN, was detected in the distal region on the short arm of chromosome I (Fig. 2a). After 8 h of light, this genomic region also accounted for the most significant contribution to leaf sucrose content and explained 22.3 % of the phenotypic variance (Fig. 2b).

Tuberization onset involves many changes in metabolism and carbohydrate partitioning in potato plants (Viola et al. 2001; Ferreira et al. 2010). Šimko et al. (1999) analysed the relationship between the rate of export of sugars from leaves and earliness of tuberization in potato. They mapped QTL for sugar concentrations in phloem sap collected from potato leaves at the end of a light period and identified three QTL for sucrose content, of which the major effect QTL, suc8.1, was located on chromosome VIII. Altogether, they found that three QTL accounted for as much as 26.9 % of the sucrose content variance they observed. In the present study, we identified six and three QTL for leaf sucrose content in 11-week-old population 12–3 plants after dark and light periods, respectively (Table 2). Altogether, the corresponding total effects of the QTL-tagging marker alleles explained approximately 76 and 37 % of the observed phenotypic variances, respectively.

A *Cycling DOF Factor1* (*CDF1*) gene located on potato chromosome V is potentially associated with regulation of photoperiodic tuberization in potato through modulating the carbon flux (Kloosterman et al. 2013; Shan et al. 2013). In the present study, the most significant QTL accounting for 15.8 % of the variance in leaf sucrose content in 11-week-old plants after dark phase (Table 2; Supplementary Table S3) might contain the *CDF1* gene. The α-glucan water dikinase (GWD) activity is necessary for night degradation of transitory starch in potato leaves (Lloyd et al. 2005; Hofius and Börnke 2007). The *GWD* gene was mapped on chromosome V (Werij et al. 2012; Schreiber et al. 2014). In population 12–3, the peak of the QTL LSC11AN corresponds to the marker pPt-656044 position (Table 2). It is worth noting that the marker pPt-656044 and the *GWD* locus were separated by a distance of ca. 10 cM on the genetic map PGSC *S. tuberosum* group Phureja DM1-3 (DM1-3) (Sharma et al. 2013). *GWD* was not mapped in population 12–3. However, it is likely located outside the QTL LSC11AN and not contributing to its effect.

In 11-week-old plants after the light period, LSC11AL on chromosome I was the most significant QTL, explaining 15.5 % of the observed variance in leaf sucrose content (Fig. 2d), and was found to co-localize with LSC11AN (Fig. 2c). The marker capPt-673196 from the LSC11AN/LSC11AL peak has been reported to be located on chromosome 1 of the DM1-3 genome (i.e. chr01 72360189.0.72360700, v4.03; Supplementary Table S3) close to the *BMY*-*1* locus (chr01 72104900.0.72114100; Schreiber et al. 2014; Supplementary Table S3). The genetic distance between the marker capPt-673196 and *BMY*-*1* is 0–2 cM (Sharma et al. 2013). A prior study of transgenic potato plants demonstrated the significance of β-amylase activity in transitory starch mobilization (Scheidig et al. 2002). Therefore, it is our view that *BMY*-*1* may underlie the QTL for LSC11AN and LSC11AL on chromosome I. Genes encoding two other amylases mapped in this study were both localized within QTL regions. Marker NL-AAM, derived from a sequence of α-amylase (Ren et al. 2007), was mapped onto chromosome I and had a significant effect on LSC5AL (*R*^2^ = 15.9 %). Marker β-AmyI, designed on the basis of the sequence encoding a plastidic β-amylase, was found to co-localize with a QTL for tuber starch content on chromosome VIII that 12.8 % of the variance in this trait could be ascribed to (Supplementary Table S1). A putative role for β-amylase in the accumulation of reducing sugars in potato tubers has been suggested previously (Krusiewicz et al. 2011); β-amylase activity might also affect tuber starch content.

Sucrose produced in potato leaves is the main carbon compound used in transitory starch synthesis in leaves and stored as starch in tubers. However, photosynthetic and non-photosynthetic cells employ different starch synthesis pathways (Bahaji et al. 2014). Although all of the major genes involved in potato tuber starch biosynthesis have been cloned (Geigenberger et al. 2004), the mechanisms involved in starch metabolism and its regulation are still unclear (Ferreira et al. 2010). Improvements in starch yield and starch quality are needed in agriculture and industrial applications, respectively (Bahaji et al. 2014). The natural variation of the genes involved in starch metabolism, even within plants of the same species, may result in variability in synthesis of amylose and amylopectin, as well as differences in the structure and properties of the starches formed (Ellis et al. 1998). For example, amylose content in potato tubers is affected by the composition of alleles encoding granule-bound starch synthase I (Van de Wal et al. 2001). Tuber starch content and quality also depend on the source of the introgressed alleles affecting these traits. Jansen et al. (2001) concluded that the wild potato species *S. chacoense* stands out as especially suitable for breeding of potatoes with high starch content. The theoretical contributions of *S. chacoense* in the origin of the parental clones used in the present study, DG 00-683 and DG 08-28/13, were 28 and 11 %, respectively. We cannot exclude that *S. chacoense* and/or other wild *Solanum* species being in pedigree of these clones had a strong influence on the starch content of tubers in our population 12–3.

Schäfer-Pregl et al. (1998) detected eighteen QTL for starch content on all twelve potato chromosomes. In other linkage studies, from four (McCord et al. 2011) to five (Freyre and Douches 1994b; Werij et al. 2012) QTL for starch content/specific gravity have been mapped in potato. We were able to identify twelve QTL for this trait on seven potato chromosomes: I, II, III, VIII, X, XI, and XII. The most important QTL was mapped to the chromosome I region spanning 63 cM, in which the map position of *AGPaseS*-*a* was reported. AGPase converts G1P and ATP into PPi and ADP-glucose (Geigenberger 2011; Sonnewald and Kossmann 2013) and is the sole source of ADP-glucose for starch biosynthesis in heterotrophic organs (Bahaji et al. 2014). Five loci for two subunits of AGPase, S and B, were mapped to chromosomes I, IV, VII, VIII and XII in the potato genome (Chen et al. 2001). *AGPaseS* is one of the three expressed genes encoding the large subunit of this enzyme in the potato genome (Schreiber et al. 2014). Large AGPase subunits are critical for the enzyme’s allosteric properties (Georgelis et al. 2009). Former studies of experimental mapping populations indicated a small effect of *AGPaseS*-*a* on this trait (Schäfer-Pregl et al. 1998; Gebhardt et al. 2005). In association studies, the amplicons AGPsS-9a and AGPsS-10a, both derived from the *AGPaseS*-*a* locus, correlated either positively or negatively with tuber starch content (Li et al. 2013). In our study, a significant difference in *AGPaseS*-*a* expression was observed between the parental clones with high versus low tuber starch content. The results obtained from our examination of 20 progeny plants indicate that higher *AGPaseS*-*a* expression coincides with the presence of the marker allele AGPaseS-a_1334_ (Fig. 3). Moreover, *AGPaseS*-*a* allelic diversity was related to differing levels of *AGPaseS*-*a* expression and the *AGPaseS*-*a* gene co-localized with a QTL for tuber starch content. These findings suggest that AGPaseS-a activity may be regulated at the transcription level and that its expression level affected the starch content of the tubers in population 12–3. The *AGPaseS*-*a* allele contributed significantly to, but was not necessary for, high tuber starch content (LOD = 6.56, *R*^2^ = 15.2 %, Fig. 3; Supplementary Table S1. Tuber starch content was affected by other QTL; both high (bulk II) and low (bulk III) starch phenotypes were observed in the absence and presence of the *AGPaseS*-*a* allele, respectively (Fig. 3).

We found that seven QTL for tuber starch content mapped to similar genetic positions as QTL for leaf sucrose content. Overlapping of QTL for different traits evaluated in different organs does not prove that the observed phenotypes result from pleiotropic effects of a single gene, as opposed to resulting from effects of closely linked but unrelated genes (Gebhardt et al. 2005). Our results provide knowledge for further research examining source-sink interactions and relationships between allelic variants of genes that influence carbon transition from potato leaves and the genes that underlie starch biosynthesis in tubers.

### **Author contribution statement**

JŚ constructed the genetic map, conducted the QTL analysis and co-wrote the manuscript. DSK conducted phenotyping, detected CAPS/SCAR markers and contributed to manuscript writing. KS performed the AGPase expression studies, the marker screening and cloning. JWS undertook crossing. DSŻ oversaw the plant material. EZG contributed to selection of the parental clones. HJ oversaw development of the mapping population and phenotyping experiments. WM conceived and coordinated the project, and co-wrote the manuscript.

## Electronic supplementary material

Supplementary material 1 (DOCX 77 kb)

Supplementary material 2 (XLSX 14 kb)

Supplementary material 3 (DOCX 22 kb)

Supplementary material 4 (DOCX 22 kb)
