# Supplementary material for: Mapping of quantitative trait loci for tuber starch and leaf sucrose contents in diploid potato
Source: Theor Appl Genet. 2015 Oct 14;129:131–40. doi: 10.1007/s00122-015-2615-9 (PMC4703618; doi:10.1007/s00122-015-2615-9)
Supplement: Supplementary file 1 — Supplementary material 1 (DOCX 77 kb) [file 122_2015_2615_MOESM1_ESM.docx]

**Mapping of quantitative trait loci for tuber starch and leaf sucrose contents in diploid potato**

Jadwiga Śliwka, Dorota Sołtys-Kalina, Katarzyna Szajko, Iwona Wasilewicz-Flis, Danuta Strzelczyk-Żyta, Ewa Zimnoch-Guzowska, Henryka Jakuczun, Waldemar Marczewski*

Plant Breeding and Acclimatization Institute – National Research Institute, Młochów, Platanowa 19, 05-831 Młochów, Poland

*Corresponding author: Waldemar Marczewski; [w.marczewski@ihar.edu.pl](mailto:w.marczewski@ihar.edu.pl)


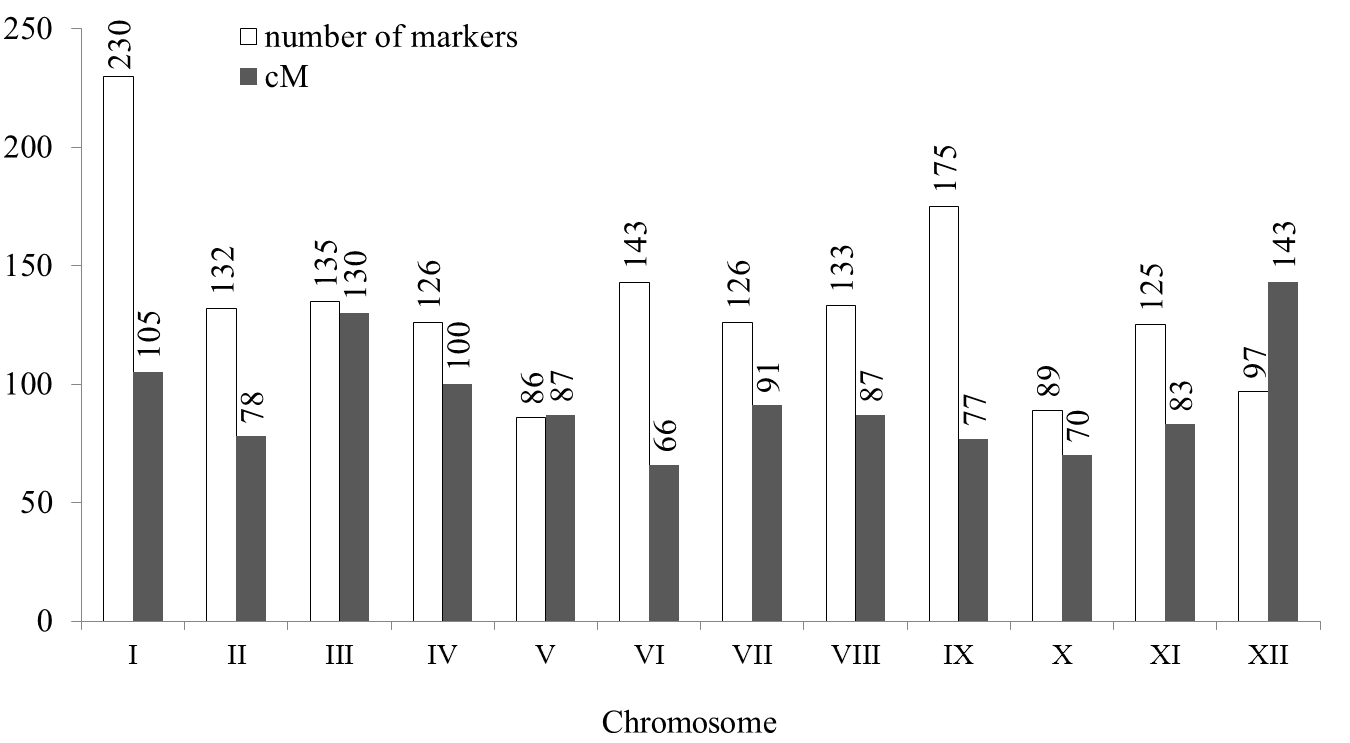


**Supplementary Fig. S1** Distribution of markers and length of chromosomes of the genetic map constructed for mapping population 12-3. The map was produced in JoinMap® 4 (Van Ooijen 2006).
