# Supplementary material for: Mapping of quantitative trait loci for tuber starch and leaf sucrose contents in diploid potato
Source: Theor Appl Genet. 2015 Oct 14;129:131–40. doi: 10.1007/s00122-015-2615-9 (PMC4703618; doi:10.1007/s00122-015-2615-9)
Supplement: Supplementary file 3 — Supplementary material 3 (DOCX 22 kb) [file 122_2015_2615_MOESM3_ESM.docx]

**Mapping of quantitative trait loci for tuber starch and leaf sucrose contents in diploid potato**

Jadwiga Śliwka, Dorota Sołtys-Kalina, Katarzyna Szajko, Iwona Wasilewicz-Flis, Danuta Strzelczyk-Żyta, Ewa Zimnoch-Guzowska, Henryka Jakuczun, Waldemar Marczewski*

Plant Breeding and Acclimatization Institute – National Research Institute, Młochów, Platanowa 19, 05-831 Młochów, Poland

*Corresponding author: Waldemar Marczewski; [w.marczewski@ihar.edu.pl](mailto:w.marczewski@ihar.edu.pl)

**Supplementary Table S2** QTL detected for tuber starch content in years 2012-2014 of the diploid potato population 12-3. Interval mapping of QTL was performed using MapQTL ® 6 (Van Ooijen 2009)

| Chromosome | Marker/interval^1^ | Marker origin^2^ | Trait | Position  (cM) | LOD | *R^2^* (%) |
| --- | --- | --- | --- | --- | --- | --- |
| I |  | P2 | TSC12 | 3.5 | 3.40 | 8.2 |
|  |  |  | TSC13 |  | 3.22 | 8.0 |
|  |  |  | TSC14 |  | 3.26 | 8.1 |
|  | capPt-673196 | P1 | TSC12 | 63.0 | 5.97 | 13.9 |
|  |  |  | TSC13 |  | 7.47 | 17.5 |
|  |  |  | TSC14 |  | 6.46 | 15.3 |
|  | toPt-440651 | H | TSC12 | 84.0 | 5.51 | 13.0 |
|  |  |  | TSC13 |  | 8.09 | 18.8 |
|  |  |  | TSC14 |  | 7.89 | 18.4 |
|  |  |  |  |  |  |  |
| II | pPt-656098 | P2 | TSC12 | 56.0 | 3.87 | 9.3 |
|  | pPt-552441 | P2 | TSC13 | 56.0 | 3.83 | 9.4 |
|  |  |  | TSC14 |  | 3.02 | 7.5 |
|  |  |  |  |  |  |  |
| III |  | P2 | TSC12 | 68.1 | 3.38 | 8.2 |
|  |  |  | TSC14 |  | 3.38 | 8.3 |
|  |  |  |  |  |  |  |
| VIII | pPt-652452 | H | TSC12 | 32.8 | 3.24 | 7.8 |
|  | pPt-656209 | P1 | TSC12 | 34.8 | 3.34 | 8.1 |
|  | toPt-438845 | H | TSC12 | 39.6 | 6.08 | 14.2 |
|  |  |  | TSC13 |  | 3.49 | 8.6 |
|  |  |  | TSC14 |  | 4.84 | 11.7 |
|  |  |  |  |  |  |  |
| X | pPt-651091 | P1 | TSC12 | 24.5 | 4.28 | 10.2 |
|  |  |  | TSC13 |  | 3.24 | 8.0 |
|  |  |  | TSC14 |  | 5.02 | 12.1 |
|  | pPt-533878 | P1 | TSC12 | 28.8 | 5.06 | 12.0 |
|  |  |  | TSC13 |  | 6.10 | 14.5 |
|  |  |  | TSC14 |  | 5.17 | 12.5 |
|  |  |  |  |  |  |  |
| XI | pPt-471789 | H | TSC12 | 54.1 | 3.90 | 9.3 |
|  |  |  | TSC13 |  | 3.60 | 8.8 |
|  |  |  | TSC14 |  | 5.56 | 13.3 |
|  |  |  |  |  |  |  |
| XII |  | P2 | TSC12 | 46.3 | 4.22 | 10.1 |
|  | pPt-656237 | P1 | TSC14 | 143.5 | 5.25 | 12.6 |
|  |  |  |  |  |  |  |

^1^ in case of intervals without a marker, origins of the flanking markers are given. The software divides linkage groups into theoretical intervals that in case of less dense map segments may not contain a marker.

^2^P1 - inherited from DG 00-683; P2 - inherited from DG 08-28/13; H – heterozygous in both parents;
