# Supplementary material for: Mapping of quantitative trait loci for tuber starch and leaf sucrose contents in diploid potato
Source: Theor Appl Genet. 2015 Oct 14;129:131–40. doi: 10.1007/s00122-015-2615-9 (PMC4703618; doi:10.1007/s00122-015-2615-9)
Supplement: Supplementary file 4 — Supplementary material 4 (DOCX 22 kb) [file 122_2015_2615_MOESM4_ESM.docx]

**Mapping of quantitative trait loci for tuber starch and leaf sucrose contents in diploid potato**

Jadwiga Śliwka, Dorota Sołtys-Kalina, Katarzyna Szajko, Iwona Wasilewicz-Flis, Danuta Strzelczyk-Żyta, Ewa Zimnoch-Guzowska, Henryka Jakuczun, Waldemar Marczewski*

Plant Breeding and Acclimatization Institute – National Research Institute, Młochów, Platanowa 19, 05-831 Młochów, Poland

*Corresponding author: Waldemar Marczewski; [w.marczewski@ihar.edu.pl](mailto:w.marczewski@ihar.edu.pl)

**Supplementary Table S3** Physical location of QTL detected using diploid potato population 12-3 in comparison to locations of the candidate genes involved in carbohydrate metabolism. QTL for: tuber starch content (TSC, means 2012-14) and leaf sucrose content (LSC) measured after 8 h of darkness (AN) or light (AL) in 5- and 11-week-old plants.

| Chromo­some | Trait | Significant interval (cM*)* | Flanking markers | Position of the marker in DM1-3 v4.03 potato genome | Candidate gene and its location (based on Schreiber et al. 2014; Kloosterman et al. 2013^1^) | |
| --- | --- | --- | --- | --- | --- | --- |
| I | LSC5AN | 0-13.2 | pPt-536705-  pPt-656788 | Unknown  chr00:24867095..24867166 |  |  |
|  | LSC5AL | 0-15.6 |  |  |  |  |
|  | TSC | 0-12.3 |  |  |  |  |
|  | LSC5AL | 22.2-49.01,  61.3-67.6 | pPt-652532-  pPt-534735  pPt-471360-  pPt-558970 | Unknown  Unknown  chr01:72208169..72209024  chr01:72051242..72051829 | *AMY‐1*  *AGPaseS‐1/1*  *HT‐1* | chr01:59361270..59364450  chr01:62144770..62149140  chr01:63002770..63005240 |
|  | LSC11AN | 57.5-71.5 | pPt-535462-  pPt-536916 | chr01:70410943..70411368  Unknown |  |  |
|  | LSC11AL | 57.5-72.8 | pPt-535462-  pPt-655579 | chr01:70410943..70411368  chr01:74928618..74929823 | *BMY‐1* | chr01:72104900..72114100 |
|  | TSC | 42.0-104.63 | pPt-459005-  pPt-458015 | chr01:69082743..69083253  chr01:87950287..87950946 | *INV‐1/2*  *AGPaseS‐1/2*  *INV‐1/3* | chr01:78430100..78436100  chr01:86092270..86097270  chr01:87160540..87164480 |
| II | LSC11AN | 55.9-56.0 | pPt-471594-  pPt-656098 | chr02:31679395..31679860  Unknown |  |  |
|  | TSC | 35.0-56.0 | pPt-471994-  pPt-552441 | Unknown  chr02:31827082..31827576 | *SS IV* | chr02:30142700..30152300 |
| III | TSC | 65.1-72.1 | toPt-437014-  pPt-538033 | chr03:43864148..43864596  chr01:83890064..83890882 | *SssI*  *SUS‐3*  *KT‐InvInh‐3/2*  *SEX4*  *HXK‐3*  *INV‐cw‐3* | chr03:45887500..45896600  chr03:48949000..48950010  chr03:49448370..49449150  chr03:50875700..50885600  chr03:60521600..60527100  chr03:61076900..61079820 |
| V | LSC11AN | 22.0-25.5 | pPt-457785-  pPt-651719 | Unknown  chr05:6493158..6493618 | *GWD*  *CDF^1^* | chr05:9823400..9839000 chr05:4538880..4541736 |
| VIII | LSC11AL | 31.6-32.8 | pPt-472947-  pPt-472276 | Unknown  chr08:45990936..45991280 |  |  |
|  | LSC11AN | 34.8 | pPt-656209 | Unknown |  |  |
|  | TSC | 22.8-39.6 | pPt-457053-  toPt-438845 | Unknown  chr08:47200986..47201346 |  |  |
| IX | LSC5AL | 51.7-51.9 | pPt-657361-  pPt-538393 | chr09:44868524..44869333  chr09:44032306..44033182 | *SBE II* | chr09:3737100..3755900 |
|  | LSC11AL | 52.9 | pPt-537372 | chr09:33741465..33741956 |  |  |
| X | LSC5AL | 24.3-25.5 | pPt-535571-  pPt-538196 | chr10:4444710..4444960  chr10:4449746..4450101 |  |  |
|  | LSC11AN | 27.1 | pPt-552383 | chr05:13288091..13288552 |  |  |
|  | TSC | 16.5-46.5 | pPt-473473-  pPt-539969 | chr10:27044463..27045342  chr10:54207366..54207982 | *DBE‐10 INV‐*  *InvInh‐10/2*  *InvInh‐10/3*  *10/2*  *INV‐10/3* | chr10:38081140..38083210  chr10:50545200..50545720  chr10:51072890..51073410  chr10:53235940..53238050  chr10:53706680..53710620 |
| XI | TSC | 49.8-55.8 | pPt-456680-  pPt-456531 | Unknown  Unknown |  |  |
| XII | LSC11AN | 38.5-49.6 | pPt-536168  pPt-657438 | chr12:8576485..8577047  Unknown |  |  |
|  | TSC | 117.6-143.5 | pPt-456625  pPt-656237 | Unknown  chr02:41748825..4174915 |  |  |
|  |  |  |  |  |  |  |

­­­­­­­­­­­­­­­­­
